# Supplementary material for: Study for Evaluation of Hydrogels after the Incorporation of Liposomes Embedded with Caffeic Acid
Source: Pharmaceuticals (Basel). 2022 Jan 31;15(2):175. doi: 10.3390/ph15020175 (PMC8875116; doi:10.3390/ph15020175)
Supplement: Supplementary file 1 [file pharmaceuticals-15-00175-s001.zip › pharmaceuticals-1527890-supplementary.pdf]

pharmaceuticals

Article

Study for Evaluation of Hydrogels after the Incorporation of Liposomes Embedded with Caffeic Acid

Ioana Lavinia Dejeu [1], Laura Grațiela Vicaș [2,*], Lavinia Lia Vlaia [3,*], Tunde Jurca [2], Mariana Eugenia Mureșan [2], Annamaria Pallag [2], Georgeta Hermina Coneac [3], Ioana Viorica Olariu [3], Ana Maria Muț [3], Anca Salomea Bodea [1], George Emanuiel Dejeu [2,†], Octavian Adrian Maghiar [2] and Eleonora Marian [2]

[1] Doctoral School of Biomedical Science, University of Oradea, 1 University Street, 410087 Oradea, Romania; ioana.dejeu@gmail.com (I.L.D); salomea.bodea@gmail.com (A.S.B.)
[2] Faculty of Medicine and Pharmacy, University of Oradea, 1 Decembrie Street, 410073 Oradea, Romania; jurcatunde@yahoo.com (T.J.); marianamur2002@yahoo.com (M.E.M.); annamariapallag@gmail.com (A.P.); dejeu.george@gmail.com (G.E.D); octimaghiar@gmail.com (O.A.M.); marian_eleonora@yahoo.com (E.M.)
[3] Department II—Pharmaceutical Technology, Formulation and Technology of Drugs Research Center, ”Victor Babeș” University of Medicine and Pharmacy, Eftimie Murgu Sq. no. 2, 300041 Timișoara, Romania; coneac.georgeta@umft.ro (G.H.C.); olariu.ioana@umft.ro (I.V.O.); mut.anamaria@umft.ro (A.M.M.)
* Correspondence: vlaia.lavinia@umft.ro (L.L.V.); laura.vicas@gmail.com (L.G.V.)
† These authors contributed equally to this work.

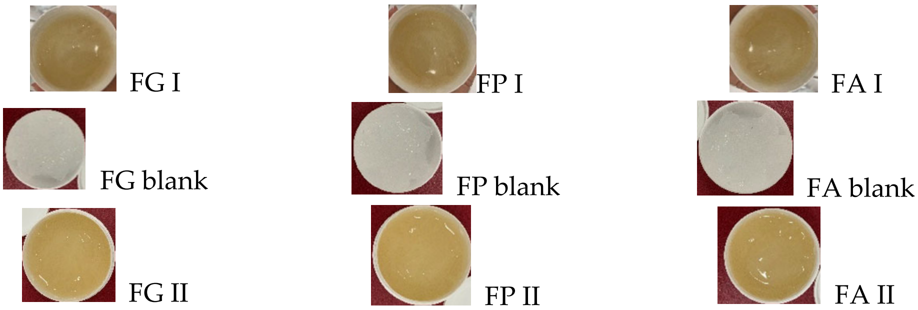

Figure S1. The color of hydrogels.

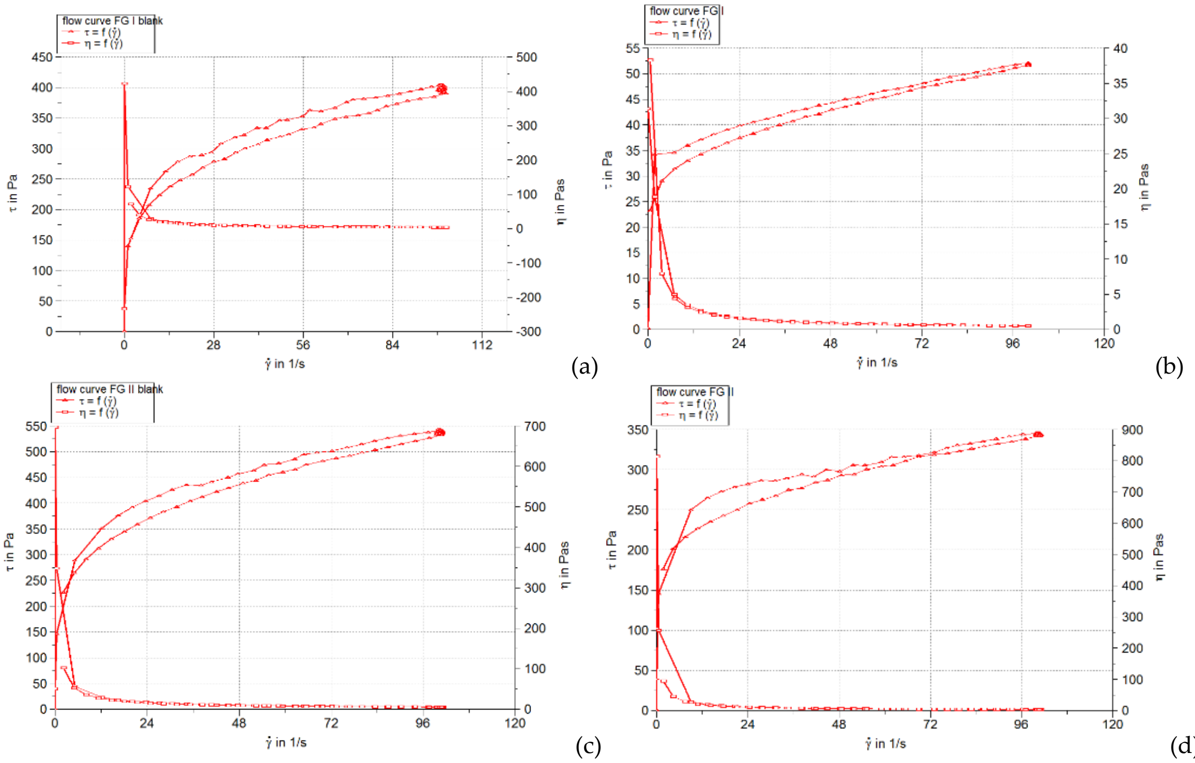

Figure S2. Rheograms and viscosity curves of Carbopol 940 and glycerol hydrogels, simple or loaded with caffeic acid liposomes: (a) FGI blank, (b) FGI, (c) FGII blank, (d) FGII.

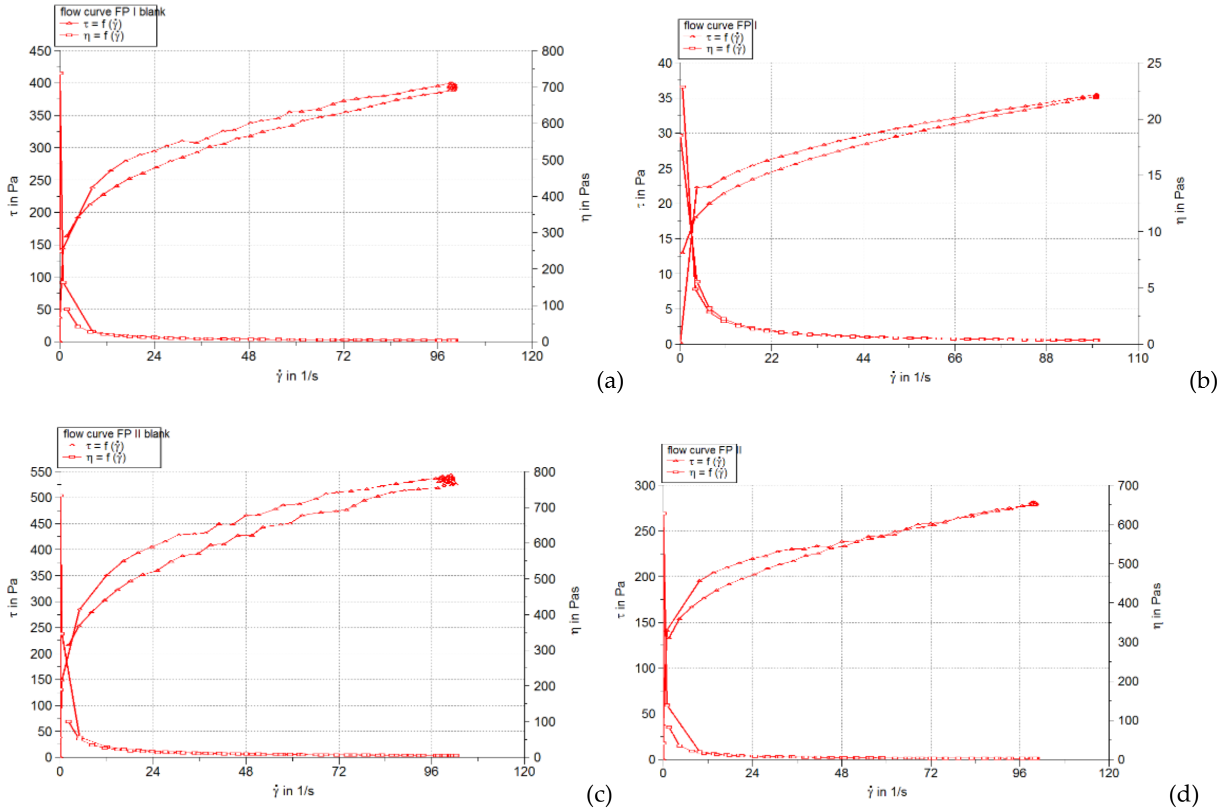

Figure S3. Rheograms and viscosity curves of Carbopol 940 and propylene glycol hydrogels, simple or loaded with caffeic acid liposomes: (a) FPI blank, (b) FPI, (c) FPII blank, (d) FPII.

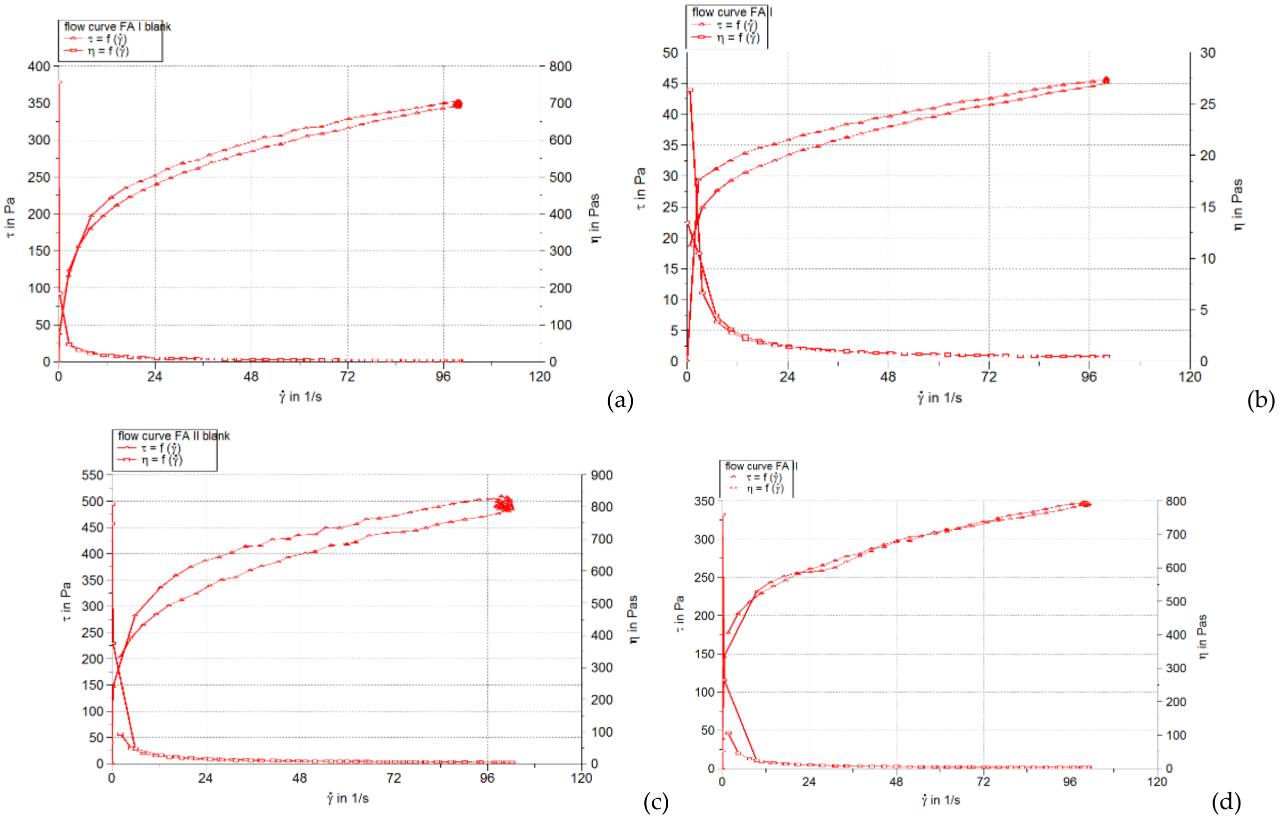

Figure S4. Rheograms and viscosity curves of Carbopol 940 and isopropyl alcohol hydrogels, simple or loaded with caffeic acid liposomes: (a) FAI blank, (b) FAI, (c) FAII blank, (d) FAII.

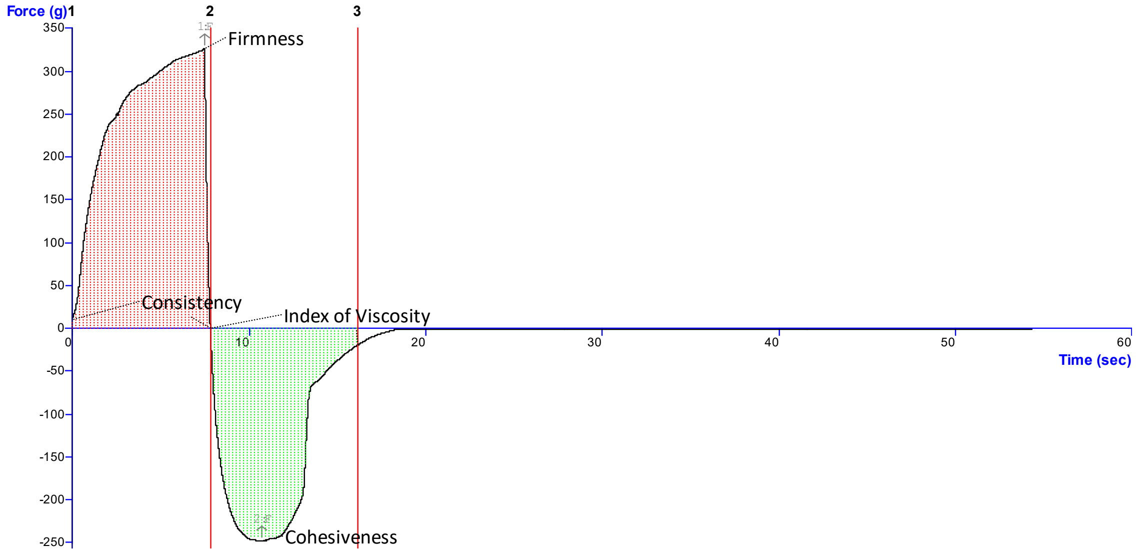

Figure S5. Typical force versus time profile and textural parameters determined by the back compression-extrusion test for Carbopol 940 based experimental hydrogels.

Table S1. The percent of CA released in time (h) from hydrogels, expressed as mean ± standard deviation.

Time (h)	CA (%)	FG I (%)	FP I (%)	FA I (%)	FG II (%)	FP II (%)	FA II (%)
0.5	10.32±0.21	3.05±0.13	2.44±0.09	2.64±0.08	2.50±0.11	2.45±0.09	1.65±0.11
1	24.24±0.11	11.29±0.22	10.38±0.24	10.12±0.29	12.66±0.26	12.06±0.20	11.51±0.09
2	54.88±0.18	24.16±0.41	23.35±0.11	22.58±0.41	21.72±0.38	21.12±0.30	20.42±0.037
3	71.96±1.25	34.38±0.54	32.76±0.33	33.93±0.88	31.78±0.58	31.38±0.51	30.43±0.70
4	91.32±1.30	45.62±1.10	44.35±1.14	44.86±0.97	39.99±0.81	38.84±0.59	38.04±0.82
5	92.68±2.92	55.34±1.57	53.41±1.73	54.22±1.91	52.25±1.89	51.20±0.88	50.10±1.19
6	92.88±3.12	69.07±2.20	68.21±2.01	67.39±2.33	61.61±2.11	61.26±1.52	60.01±2.28
7	93.00±2.87	78.69±2.55	77.36±1.99	77.97±2.21	71.72±1.99	70.67±2.19	69.52±2.33
8	93.12±1.98	89.27±3.01	87.69±2.57	88.40±2.49	77.13±2.37	76.31±2.26	76.03±2.40
12	93.24±3.42	88.91±3.71	88.25±3.41	88.76±2.52	86.29±2.82	86.19±2.33	84.98±2.51
